# A radiomic approach for adaptive radiotherapy in non-small cell lung cancer patients

**DOI:** 10.1371/journal.pone.0207455

**Published:** 2018-11-21

**Authors:** Sara Ramella, Michele Fiore, Carlo Greco, Ermanno Cordelli, Rosa Sicilia, Mario Merone, Elisabetta Molfese, Marianna Miele, Patrizia Cornacchione, Edy Ippolito, Giulio Iannello, Rolando Maria D’Angelillo, Paolo Soda

**Affiliations:** 1 Radiotherapy Unit, Campus Bio-Medico University, Rome, Italy; 2 Computer Science and Bioinformatics Laboratory, Integrated Research Centre, Campus Bio-Medico University, Rome, Italy; University of South Alabama Mitchell Cancer Institute, UNITED STATES

## Abstract

The primary goal of precision medicine is to minimize side effects and optimize efficacy of treatments. Recent advances in medical imaging technology allow the use of more advanced image analysis methods beyond simple measurements of tumor size or radiotracer uptake metrics. The extraction of quantitative features from medical images to characterize tumor pathology or heterogeneity is an interesting process to investigate, in order to provide information that may be useful to guide the therapies and predict survival. This paper discusses the rationale supporting the concept of radiomics and the feasibility of its application to Non-Small Cell Lung Cancer in the field of radiation oncology research. We studied 91 stage III patients treated with concurrent chemoradiation and adaptive approach in case of tumor reduction during treatment. We considered 12 statistics features and 230 textural features extracted from the CT images. In our study, we used an ensemble learning method to classify patients’ data into either the adaptive or non-adaptive group during chemoradiation on the basis of the starting CT simulation. Our data supports the hypothesis that a specific signature can be identified (AUC 0.82). In our experience, a radiomic signature mixing semantic and image-based features has shown promising results for personalized adaptive radiotherapy in non-small cell lung cancer.

## Introduction

According to the National Institute of Health (NIH) definition, precision medicine refers to new prevention and treatment strategies that take individual variability into account; it is a method based on understanding of individual genes, environment and life-style [1].

Precision medicine has been introduced into routine clinical care to minimize iatrogenic damage and reach an optimal therapeutic effect [2]. The possibility to achieve this result is strictly related to modern technologies such as genomics, proteomics and radiomics because they identify the “biomarkers”, characteristics that are objectively measured and evaluated as indicators of normal biological processes, pathogenic processes or pharmacologic responses to a therapeutic intervention. In the last years, much of the discussion regarding personalized medicine has focused on molecular characterization using genomic and proteomic technologies. As these need to acquire tissue samples through invasive approaches, and often these samples are only a small portion of heterogeneous lesions, they may not accurately represent the lesion’s anatomic, functional and physiologic properties. This limits the use of biopsy based molecular assays, but in contrast it provides a huge potential for non-invasive imaging techniques which take into account the entire volume of disease [3]. Recent advances in medical imaging technology allow the use of more advanced image analysis methods beyond simple measurements of tumor size or radiotracer uptake metrics. Radiomics is the extraction of quantitative features (Quantitative Biomarkers) from medical images to characterize tumor pathology or heterogeneity (phenotype). It is an emerging field of quantitative imaging that aims to extract quantitative data from medical images to characterize tumor pathology or heterogeneity using a large set of advanced imaging features [4]. The goal of radiomics is to provide information that can be used to predict survival, as a prognostic marker, but more interestingly to guide treatment thanks to its predictive value. The possibility of predicting response to a treatment would allow for re-adaptation or intensification of therapy for the patient, in order to offer him greater chances of better outcome, at the aim to change his prognosis.

Radiomics has several implications in lung cancer. There is incontrovertible evidence for intra-tumoral heterogeneity on lung CT image for lung cancer patients and these heterogeneities can be captured with radiomic features. The first radiomic application explored by some papers refers to the diagnostic issue, such as the reduction of not-otherwise-specified tumor (NOS) in unclassified tumors of non-small-cell lung cancer [5] and the possibility to differentiate lepidic predominant adenocarcinoma [6]. Moreover, it was reported that somatic mutations drive distinct imaging phenotypes in lung cancer and a radiomic signatures was able to successfully discriminate between EGFR+ and EGFR- cases [7]. These artificial intelligence methods could be proposed to assist pathologists and clinicians in cases of unresectable tumors or scant biopsy materials for histological subtyping and cancer therapy.

The second point for evaluating radiomics is in the prediction of outcome. Clinical decisions for the treatment of lung cancer are largely based on patient characteristics such as performance status, stage at diagnosis and tumor histology. In metastatic non small cell lung cancer (NSCLC) patients, molecular information has brought remarkable results thanks to targeted therapies. On the other hand, in locally-advanced disease, standard treatment is concurrent chemoradiation which is not guided by molecular data in clinical practice.

Several papers have shown that the combination of clinical, genomic, and radiomic features, provides a prognostic signature for overall survival [8] or for prediction of distant metastasis in lung adenocarcinoma treated with chemoradiation [9] and with stereotactic radiation treatment (SBRT) [10–11]. Radiation therapy is by definition a personalized medicine because the anatomy is proper of each patient and dose distribution is tailored on the target volume and organs at risk. We do not know at the treatment start if that particular patient will achieve a response or not. We know from literature data that about 30–40% of patients who perform chemoradiation undergo a significant reduction of the tumor during treatment [12–17]. In this study, we investigated the feasibility of a system where the radiomic features of the patient’s initial imaging were able to predict tumor reduction during chemoradiation.

## Materials and methods

### Patient and CT imaging

We studied 91 stage III patients treated with concurrent chemoradiation. As reported in a previous prospective study of our group [18], 50 patients with stage IIIA/IIIB NSCLC were enrolled from November 2012 to July 2014 and treated with concurrent chemoradiation at radical dose with adaptive approach.

**Fig 1:**
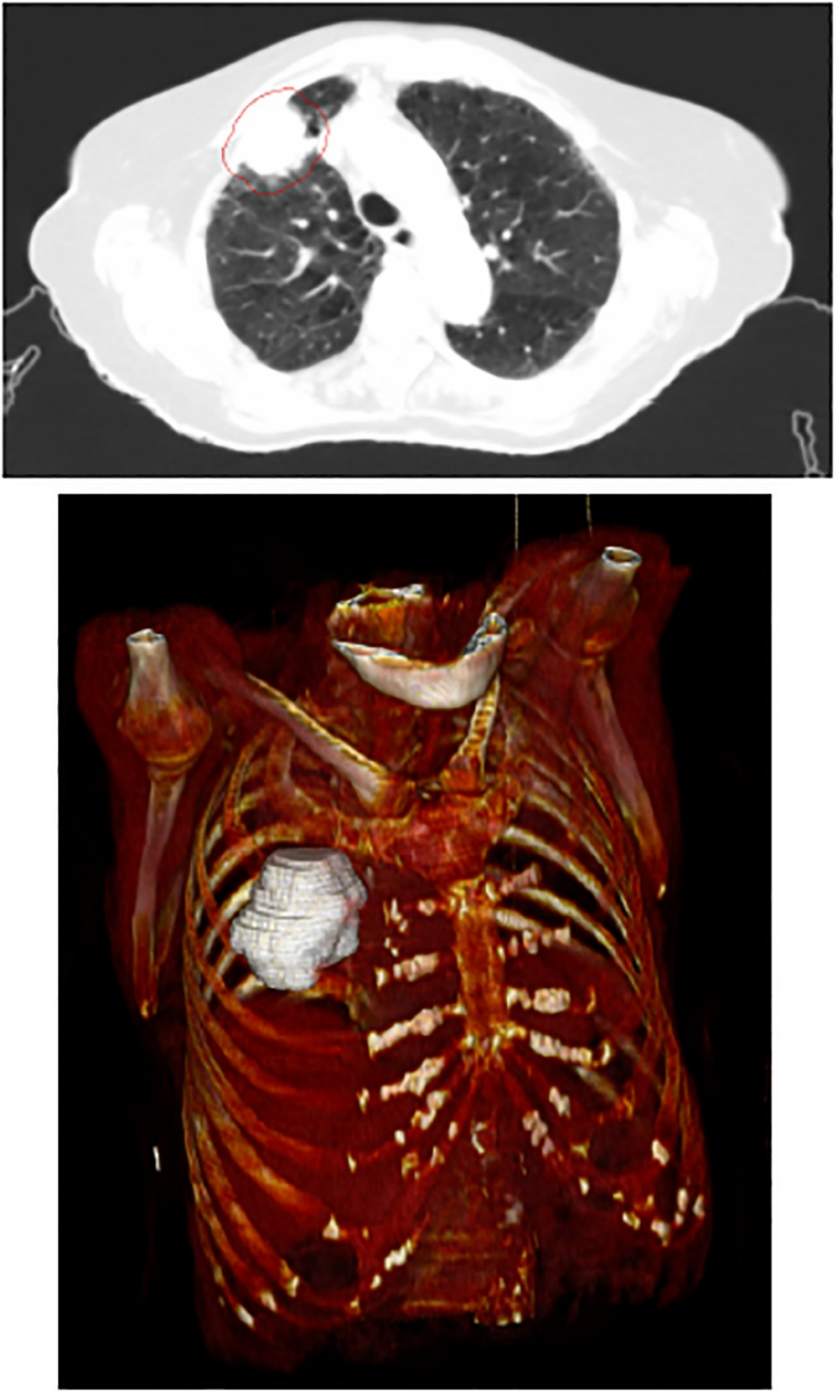
Example of ROI (Region of Interest) in 2D and 3D images for a patient in the adaptive group (lung window).

**Fig 2:**
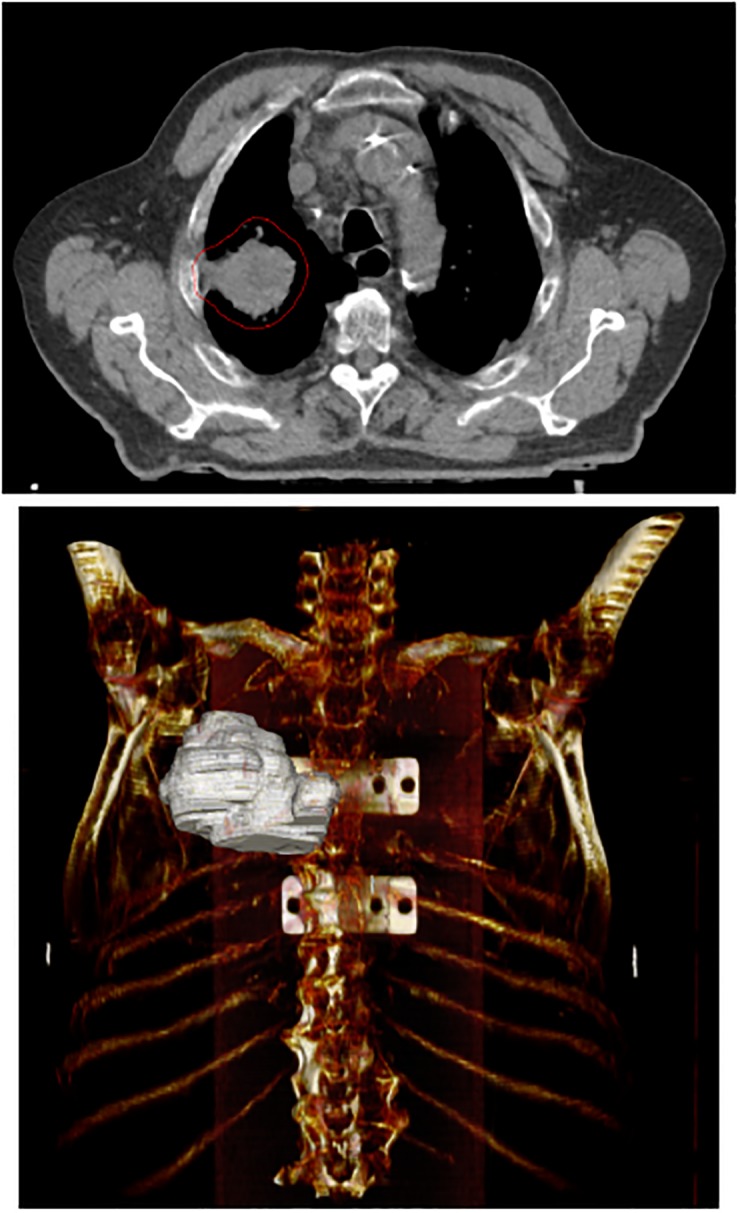
Example of ROI (Region of Interest) in 2D and 3D images for a patient in the non-adaptive group (mediastinal window).

It was defined as a reduction in tumor volume (assessed by two radiation oncologists on weekly chest CT simulations) leading to the implementation of a new treatment plan with which the patient would continue radiation therapy. Other 41 patients with the same initial characteristics (PS, stage, age, etc.) who underwent radical concurrent chemoradiation in the same period, but who did not achieve target reduction, were added to the initial group in order to investigate the predictive power of the radiomic features on tumor shrinkage (Table 1). The characteristics investigated were extracted from the initial simulation CT on which the Clinical Volume was manually delineated by expert radiation oncologists, providing a 3D ROI (Figs 1A, 1B, 2A and 2B). The adaptive protocol was approved by Ethical Committee Campus Bio-Medico University on 30 October 2012 and registered at ClinicalTrials.gov on 12 July 2018 with Identifier NCT03583723 after an initial exploratory phase.

**Table 1:** Patients’ characteristics.

	Adaptive patients (%) (n = 50)	Non-Adaptive patients (%) (n = 41)	Total (n = 91)
Age:	median: 71 years std deviation: 10.3	median: 72 years std deviation: 8.6	median: 71 years std deviation: 9.6
< 70 years	19 (38%)	18 (44%)	37 (41%)
≥ 70 years	31 (62%)	23 (56%)	54 (59%)
Sex:			
Male	39 (78%)	30 (73%)	69 (76%)
Female	11 (22%)	11 (27%)	22 (24%)
Histology:			
Adenocarcinoma	16 (32%)	23 (56%)	39 (43%)
Squamous	28 (56%)	15 (37%)	43 (47%)
NOS	3 (6%)	3 (7%)	6 (7%)
No histologic subtype available	3 (6%)	0 (0%)	3 (3%)
Stage:			
IIIA	29 (58%)	26 (63%)	55 (60%)
IIIB	21 (42%)	15 (37%)	36 (40%)
Chemo before RTCT:			
Yes	23 (46%)	28 (68%)	51 (56%)
No	27 (54%)	13 (32%)	40 (44%)
Concurrent chemo:			
Duplets	19 (38%)	20 (49%)	39 (43%)
Mono	31 (62%)	21 (51%)	52 (57%)

The Institutional review board approved this review. A written informed consent was obtained in all patients.

The authors confirm that all ongoing and related trials for this intervention are registered.

### Semantic features

Two experienced radiation oncologists (RO) independently reviewed all CT images and assigned scores to each tumor for nine semantic imaging features, divided into personal data (age, sex and smoking attitude), staging scores of the tumour (T, N and tumor stage), and histology and gene mutations evaluation.

All RO blindly assigned staging scores, in case of disagreement, they reviewed the CT images together and any discrepancy was resolved through discussion until consensus was reached.

### Radiomic feature extraction

Given each 3D ROI in the images, we computed the following radiomic features using our in-house software tool coded in MATLAB (Mathworks Inc, MA, U.S.A.), taking into consideration 12 statistics features and 230 textural features extracted from the CT images. Statistical features consist of the moments up to the fourth-order of the first-order image histogram, i.e., the mean, the standard deviation, the skewness and the kurtosis. Furthermore, the picture of grey-level distribution is also grasped by the histogram width, the energy, the entropy, the value of the histogram absolute maximum and the corresponding grey-level value, the energy around such maximum, the number of relative maxima in the histogram and their energy [19]. Texture feature are derived from the 3D gray-level co-occurrence matrix (GLCM) and from the Local Binary Patterns-TOP (LBP-TOP) [20]. The former represents the distribution of co-occurring values between neighbouring pixels according to different displacements, and its statistics correlate well with the image structure.

TOP-LBP are descriptors which assign to each pixel of the image a label comparing it with its neighbourhood matrix computed from three orthogonal planes. Histograms of LBP distributions in such planes are then concatenated.

### Radiomic feature selection

For each ROI, both semantic and radiomic features were grouped in a single array, which contains 251 features. In machine learning, as in this case, it is common to have a feature vector composed by so many elements: the rationale is that practitioners and researchers should define measures that go beyond the human interpretation of the images, as several discriminative features could be not directly mined by visual analysis. The set of features is then explored by the wrapper method to identify the most discriminative features [21]. A wrapper is a feature selection method that embeds the model hypothesis search within the feature subset search. Indeed, after defining a search procedure for all the possible feature subsets, the various subsets are generated and evaluated by training and testing a specific classification model. We selected this approach, as it is able to discover both feature dependencies, as well as to exploit the interaction between feature subset search and model selection.

In practice, we applied a leave-one-out (LOO) cross validation approach to evaluate the subsets: in this procedure at each iteration one single instance is removed from the data, creating a fold, then the model is trained on all the remaining instances and the removed instance is used for independent validation. The procedure is then iterated among all the instances of the data, processing a same number of folds.

In particular in the variable selection process, we used an LOO loop where at each iteration a wrapper method selected a subset of descriptors. To this aim, such a wrapper is based on a Random Forest and it used an inner 2-fold cross validation loop for performance evaluation. Such a procedure, therefore, returned the frequency of each feature among all the iterations of the external LOO loop. The final set of descriptors contained all the features that were selected at least in the 10% of the LOO iterations. This choice was motivated by the guidelines reported in [22] and by our prior knowledge on the problem domain.

**Fig 3:**
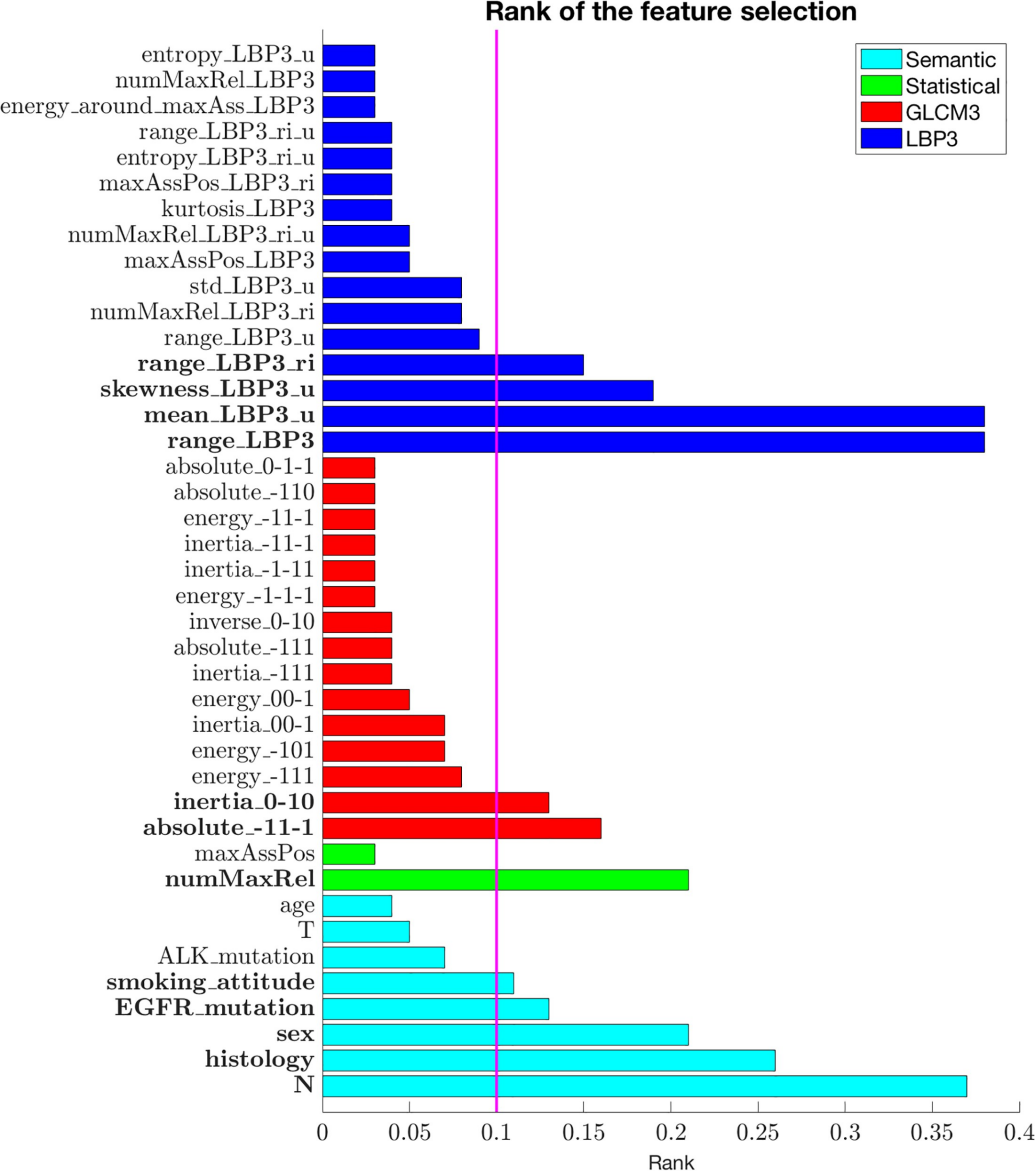
The chart shows the occurrence percentage of each feature obtained during the feature selection procedure. Due to the limited space, we report only those features selected in more than the 3% of the folds of the feature selection procedure for a total of 41 features. Moreover, we highlighted in bold style the names of the descriptors constituting the final signature. The different colors in the histogram represent the different features, as explained in the legend in the upper right corner, whilst a vertical line indicates the threshold used for defining the final signature.

After this selection procedure, five semantic features (sex, N, histology, EGFR mutation, and smoking attitude), two GLCM (“absolute_-1,1,-1” representing the textural variability in the direction given by the (-1, 1, -1) versor and “inertia_0–10” representing the textural homogeneity in the direction given by the (0, -1, 0)), four LBP-TOP measures (“range_LBP3_ri” showing the range of possible patterns in the image without considering their rotation, “skewness_LBP3_u” measuring the asymmetry of distribution of all patterns discharging noisy patterns, “mean_LBP3_u” measuring the mean pattern discharging noisy patterns ad “range_LBP3” showing the range of possible patterns in the image) and one Statistical measure (“numMaxRel” counting the number of local maximum in the histogram of the image) were included in the analysis (Fig 3).

### Classifier

In our experiments, we used a Random Forest (RF) to classify patients’ data into either the adaptive or non-adaptive group. RF is an ensemble learning method for classification that builds a multitude of decision trees at training time and provides the class that is the model of the classes of the individual trees. The number of features is denoted as p. The decision trees are built on bootstrapped training samples and, each time a split in a tree is considered, a random subset of m features is chosen, with m < p. All the experiments were conducted according to a leave-one-out cross validation, which provides a nearly unbiased estimate using only the original data, and a .632+ bootstrap validation, that on the contrary provides a measure with low variance [23].

## Results

**Fig 4:**
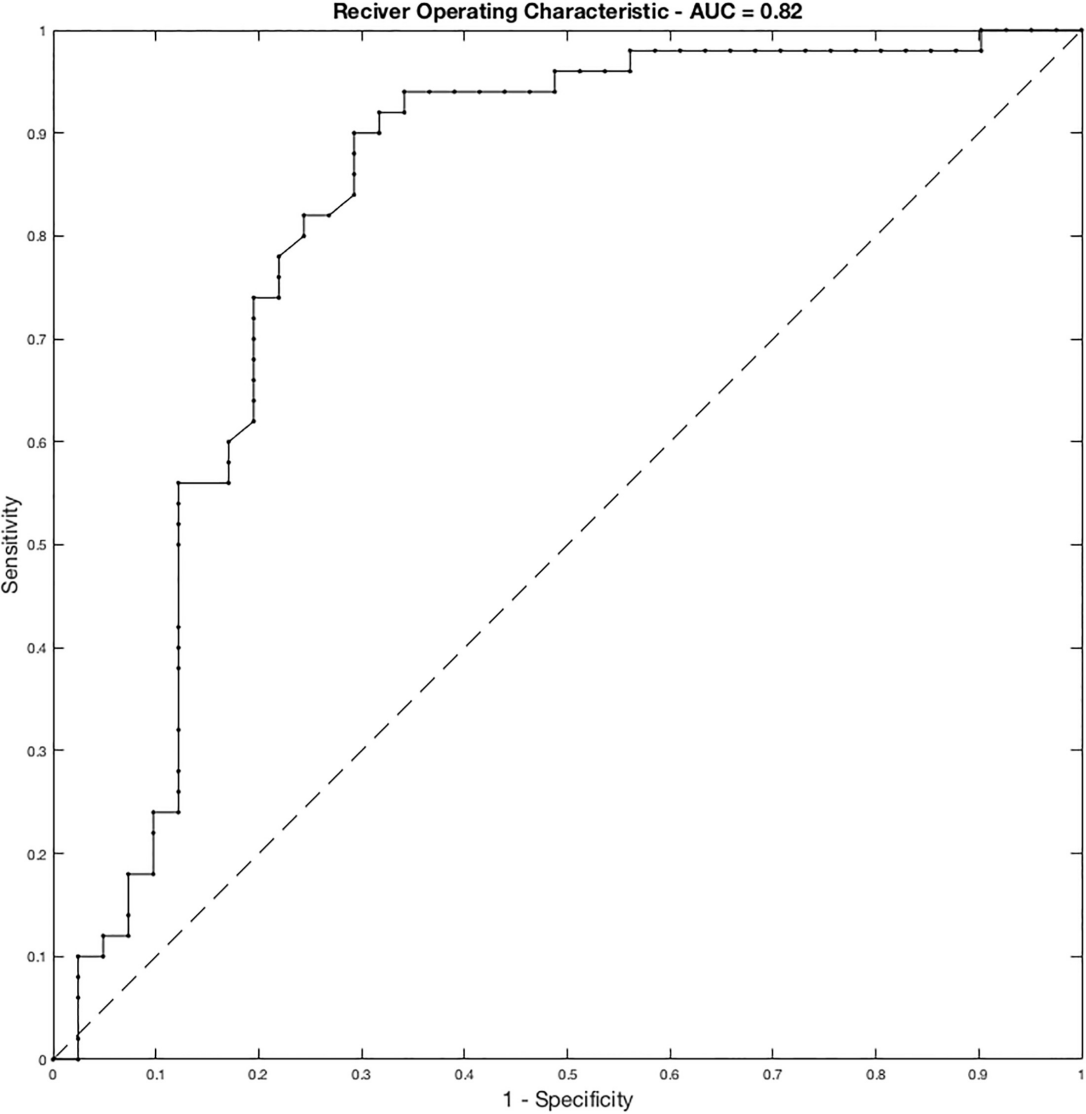
ROC curve of the proposed system.

Fig 4 displays the Receiver Operating Characteristic curve (ROC) of the proposed system, whereas Table 2 shows the results, reporting the following performance measures: the area under the receiver operating characteristic curve (AUC), the classification accuracy, the precision, the sensitivity and the Positive and Negative predictive values. Each of these metrics was computed collecting all the predictions at the end of the leave-one-out cross validation. Moreover we also reported the 95% Confidence Intervals (CIs) computed as reported in [24–26,27].

**Table 2:** Performance of the radiomic approach.

Features	AUC	Accuracy	Precision	Sensitivity	PPV	NPV
Proposed system	.820 (95% CI, 73.0% to 91.0%)	.780 (95% CI, 69.5% to 86.5%)	.778 (95% CI, 69.5% to 86.0%)	.840 (95% CI, 75.7% to 92.2%)	.657 (95% CI, 60.7% to 70.7%)	.869 (95% CI, 83.4% to 90.4%)
No semantic	.705 (95% CI, 59.6% to 81.4%)	.692 (95% CI, 59.8% to 78.7%)	.690 (95% CI, 59.7% to 78.2%)	.800 (95% CI, 70.7% to 89.2%)	.549 (95% CI, 49.7% to 60.1%)	.808 (95% CI, 76.7% to 84.%)
No GLCM	.759 (95% CI, 65.8% to 86.0%)	.736 (95% CI, 64.6% to 82.7%)	.732 (95% CI, 64.4% to 82.0%)	.820 (95% CI, 73.2% to 90.1%)	.599 (95% CI, 54.8% to 65.0%)	.841 (95% CI, 80.3% to 87.9%)
No LBP-TOP	.761 (95% CI, 66.0% to 86.1%)	.736 (95% CI, 64.6% to 82.7%)	.741 (95% CI, 65.1% to 83.1%)	.800 (95% CI, 71.0% to 89.0%)	.610 (95% CI, 55.9% to 66.1%)	.832 (95% CI, 79.3% to 87.1%)
Only semantic	.776 (95% CI, 67.8% to 87.4%)	.725 (95% CI, 63.4% to 81.7% )	.736 (95% CI, 64.4% to 82.8%)	.780 (95% CI, 68.8% to 87.2%)	.604 (95% CI, 55.3% to 65.5%)	.818 (95% CI, 77.8% to 85.8%)

While the second row of the table shows the performance achieved by the radiomic approach described hereinbefore, the following rows show what happens if some types of features are removed from the original set. In this case we repeated the same procedure explained in the previous sections for feature selection and classification; for the sake of comparison, we kept the threshold for the best subset selection equal to 10%, as before.

In particular, the third row of Table 2 reports the performance when semantic features are neglected, the fourth row shows the scores when GLCM features are discarded and the fifth row shows the scores when LBP-TOP are not used. A particular interest can be addressed to the last row where we reported the scores considering only the semantic descriptors used in clinical practice: as it is possible to notice the semantic features alone get performance which are considerably lower than the corresponding ones achieved by the radiomic signature. Indeed, using only semantic features the AUC of the system is about 4% lower than the one with the final radiomic signature, whereas the accuracy measure is about 6% lower than those of the proposed system.

For the sake of comparison, in Table 2 we also reported the Positive and Negative Predictive Values (PPV and NPV) [28] for all the experiments. It is worth noting that the PPV is considerably lower than the metrics reported in Table 2, however the Proposed system still outperforms the other experiments.

Moreover, first row of Table 3 shows the error rate computed with the bootstrap .632+, which is a method for error estimation with a lower variance than the leave-one-out cross validation [23]. The second row reports the error rate of the LOO cross validation which can be easily calculated from the accuracy measures in Table 2. Comparing the two experiments, it is clear that results with .632+ bootstrap are more biased than those computed with LOO cross validation. This could be expected since bootstrap error is usually more biased than the cross validation one, despite its lower variance [23]. It is also worth noting that the accuracies computed from bootstrap errors coherently fall in the CIs as presented in the Table 3.

**Table 3:** Leave-one-out cross validation and Bootstrap .632+ estimator errors per features set.

Errors	Proposed system	No Semantic	No GLCM	No LBP-TOP	Only Semantic
Bootstrap .632+ error	.254	.280	.278	.306	.360
Cross-validation error	.220	.308	.264	.264	.275

Finally, it is worth noticing that in all the experiments presented in Tables 2 and 3 the proposed system shows performance higher than the other experiments presented, confirming the potential and feasibility of a radiomics-based approach.

## Discussion

To the best of our knowledge, this is the first trial for feasibility and hypothesis generation of a radiomic strategy to predict tumor shrinkage during chemoradiation and our data suggests that a specific signature can be identified (AUC 0.82). Medical imaging can provide a lot of information beyond volumetric measurements and this process is referred to as image-based phenotyping. With the term phenotype refers to the set of all the characteristics manifested by a living organism, comprising its morphology, its development and its biochemical and physiological properties, including behaviour. This means that behaviour could be predicted starting from how the thing appears to us, from its image, from the phenotype. Somehow this idea seems to make our minds echo the idea of the Greek "kalòs kai agathòs", literally "beautiful and good", which are the characteristics of beauty according to the archaic Greek conception. With regard to lung cancer we should speak of ugly and bad, but also in this case, the radiomics signature computed from rountinary imaging has been unravelling tumor heterogeneity. As reported in the Introduction, this would be useful in many fields, from diagnosis [5–7] to prediction of outcome as a prognostic factor [8–11].

However, the predictive power of radiomics could be very useful for daily practice decision-making process. An example of the predictive power for radiomics application is the possibility to announce pathological response. It is a direct measure of tumor response to neoadjuvant chemoradiation assessed at time of surgery. It has the potential to be used as a surrogate endpoint for survival/local control and has been shown to be prognostic for survival in early and advanced stages for NSCLC patients. It is well known that clinical response very often is not related to pathological response after neoadjuvant therapies, due to the low value of traditional reevaluating imaging (CT and PET-TC) in this setting. A recent study investigated if pre-treatment radiomics data is able to predict pathological response after neoadjuvant chemoradiation in patients with locally advanced NSCLC [29]. One hundred and twenty-seven NSCLC patients were included in this study. Fifteen radiomic features were selected and evaluated for their power to predict pathological response. No conventional imaging features were predictive. Seven features were predictive for pathologic gross residual disease (AUC>0.6, p-value<0.05), and one for pathologic complete response (AUC = 0.63, p-value = 0.01). Tumors that did not respond well to neoadjuvant chemoradiation were more likely to present a rounder shape (AUC = 0.63, p-value = 0.009) and heterogeneous texture (AUC = 0.61, p-value = 0.03). The proven ability of radiomics to predict pathologic response on pre-treatment imaging may allow adaptation to a different therapy, if required, for those patients who may not have a complete pathological response to the initial therapy.

Results of the present study should be interpreted in the same way. Prediction of response during treatment is probably the most stimulating challenge because it would allow modifying therapy in progress. We know from literature data that about 30–40% of patients who perform chemoradiation undergo a significant reduction of the tumor during treatment [12–17]. Being able to predict this data rather than the prediction of the classical response that is obtained about a month after the end of the therapy would allow a change in therapeutic strategy, for example by intensifying the treatment itself, which would obviously not be possible once the therapy was delivered as planned at the initial time. Moreover, it would be a great advantage to know before starting chemoradiation if that particular patient is going to meet or not a tumor reduction that requires the execution of a new treatment plan, in order to optimize the workflow. Recently, also prediction using radiomics analyses of cone-beam CT images has been reported [30]. It could, therefore, be possible to modulate treatment strategy thereby offering the patient the chance to change a poor prognosis. In our experience, a radiomics signature mixing semantic and image-based features is able to predict with good performance whether a particular patient will meet or not the reduction of the target volume during chemoradiation. In future, the availability of this data before treatment could allow the specialist to intensify treatment for instance by modifying total dose, fractionation or drugs in combination with radiotherapy or even selecting consolidation therapy such as immunotherapy [31]. In the PACIFIC trial, progression-free survival was significantly longer with durvalumab than with placebo after radical chemoradiation. However, until now, no biomarkers have been identified to select patients who could benefit from this treatment that is not free from side effects. If radiomic signature is validated in future studies as a biomarker to predict response and outcome, patients at high risk for recurrence could be identified early on and become candidates for consolidation therapy. The identification of the external validation dataset is actually ongoing, even if some literature data support the use of the cross validation method [29] as applied in our work. In conclusion, the idea behind this study and the initial results obtained are certainly an original and innovative topic that opens up new research in the field of personalized medicine.

## Supporting information

S1 File Complete dataset. File containing the entire dataset of the extracted features and the labels for each patient; the “.arff” file format is the input file format for the Machine Learning software “Weka” used in the experimental process.
(ARFF)

S2 File Feature selection log file—Proposed system. Log file containing the results of the Wrapper Feature Selection procedure performed on the Complete dataset. It reports in detail the input parameters and the output ranking of the descriptors.
(TXT)

S3 File Selection log file—Semantic. Log file containing the results of the Wrapper Feature Selection procedure performed on the dataset considering only the semantic features. It reports in detail the input parameters and the output ranking of the descriptors.
(TXT)

S4 File Feature selection log file—NoSemantic. Log file containing the results of the Wrapper Feature Selection procedure performed on the dataset excluding only the semantic features. It reports in detail the input parameters and the output ranking of the descriptors.
(TXT)

S5 File Feature selection log file—NoGLCM. Log file containing the results of the Wrapper Feature Selection procedure performed on the dataset excluding only the GLCM features. It reports in detail the input parameters and the output ranking of the descriptors.
(TXT)

S6 File Feature selection log file—No LBP. Log file containing the results of the Wrapper Feature Selection procedure performed on the dataset excluding only the LBP features. It reports in detail the input parameters and the output ranking of the descriptors.
(TXT)

S7 File Performance log file—Proposed system. Log file containing the results of the performance achieved after the Classification procedure executed on the dataset containing only the final signature features for the Proposed system.
(TXT)

S8 File Performance log file—Semantic. Log file containing the results of the performance achieved after the Classification procedure executed on the dataset containing only semantic features.
(TXT)

S9 File Performance log file—NoSemantic. Log file containing the results of the performance achieved after the Classification procedure executed on the dataset containing all the features excluding the semantic descriptors.
(TXT)

S10 File Performance log file—NoGLCM. Log file containing the results of the performance achieved after the Classification procedure executed on the dataset containing all the features without GLCM descriptors.
(TXT)

S11 File Performance log file—NoLBP. Log file containing the results of the performance achieved after the Classification procedure executed on the dataset containing all the features without LBP descriptors.
(TXT)

## References

[1] Collins FS, Varmus H. A new initiative on precision medicine. New Engl J Med. 2015; 372; 793–795. 10.1056/NEJMp1500523 25635347 10.1056/NEJMp1500523 PMC5101938

[2] Aerts HJ. The Potential of Radiomic-Based Phenotyping in Precision Medicine: A Review. JAMA Oncol. 2016; 2(12):1636–1642. 10.1001/jamaoncol.2016.2631 27541161 10.1001/jamaoncol.2016.2631

[3] Burrell RA, McGranahan N, Bartek J, Swanton C. The causes and consequences of genetic heterogeneity in cancer evolution. Nature 2013;501(7467):338–45. 10.1038/nature12625 24048066 10.1038/nature12625

[4] Lambin P, Rios-Velazquez E, Leijenaar, Carvalho S, van Stiphout RG, Granton P, et al Radiomics: Extracting more information from medical images using advanced feature analysis. Eur J Cancer 2012;48:441–6. 10.1016/j.ejca.2011.11.036 22257792 10.1016/j.ejca.2011.11.036 PMC4533986

[5] Saad M, Choi TS. Deciphering unclassified tumors of non-small-cell lung cancer through radiomics. Comput Biol Med. 2017; 91:222–230. 10.1016/j.compbiomed.2017.10.029 29100116 10.1016/j.compbiomed.2017.10.029

[6] Wang H, Schabath MB, Liu Y, Berglund AE, Bloom GC, Kim J, et al Semi-quantitative Computed Tomography Characteristics for Lung Adenocarcinoma and Their Association With Lung Cancer Survival.Clin Lung Cancer. 2015;16(6):e141–63. 10.1016/j.cllc.2015.05.007 26077095 10.1016/j.cllc.2015.05.007 PMC4609593

[7] Rios Velazquez E, Parmar C, Liu Y, Coroller TP, Cruz G, Stringfield O, et al Somatic Mutations Drive Distinct Imaging Phenotypes in Lung Cancer. Cancer Res. 2017; 77(14):3922–3930. 10.1158/0008-5472.CAN-17-0122 28566328 10.1158/0008-5472.CAN-17-0122 PMC5528160

[8] Grossmann P, Stringfield O, El-Hachem N, Bui MM, Rios Velazquez E, Parmar C, et al Defining the biological basis of radiomic phenotypes in lung cancer. Elife. 2017;6 10.7554/eLife.23421 28731408 10.7554/eLife.23421 PMC5590809

[9] Coroller TP, Grossmann P, Hou Y, Rios Velazquez E, Leijenaar RT, Hermann G, et al CT-based radiomic signature predicts distant metastasis in lung adenocarcinoma. Radiother Oncol. 2015; 114(3):345–50. 10.1016/j.radonc.2015.02.015 25746350 10.1016/j.radonc.2015.02.015 PMC4400248

[10] Huynh E, Coroller TP, Narayan V, Agrawal V, Hou Y, Romano J, et al CT-based radiomic analysis of stereotactic body radiation therapy patients with lung cancer. Radiother Oncol. 2016;120(2):258–66. 10.1016/j.radonc.2016.05.024 27296412 10.1016/j.radonc.2016.05.024

[11] Li Q, Kim J, Balagurunathan Y, Qi J, Liu Y, Latifi K, et al CT imaging features associated with recurrence in non-small cell lung cancer patients after stereotactic body radiotherapy. Radiat Oncol. 2017;12(1):158 10.1186/s13014-017-0892-y 28946909 10.1186/s13014-017-0892-y PMC5613447

[12] Lim G, Bezjak A, Higgins J, Moseley D, Hope AJ, Sun A, et al Tumor regression and positional changes in non-small cell lung cancer during radical radiotherapy. J Thorac Oncol. 2011; 6: 531–536. 10.1097/JTO.0b013e31820b8a52 21258244 10.1097/JTO.0b013e31820b8a52

[13] Fox J, Ford E, Redmond K, Zhou J, Wong J, Song DY. Quantification of tumor volume changes during radiotherapy for non-small-cell lung cancer. Int J Radiat Oncol Biol Phys. 2009; 74:341–348. 10.1016/j.ijrobp.2008.07.063 19038504 10.1016/j.ijrobp.2008.07.063

[14] Kupelian PA, Ramsey C, Meeks SL, Willoughby TR, Forbes A, Wagner TH, et al Serial megavoltage CT imaging during external beam radiotherapy for non-small cell lung cancer: observations on tumor regression during treatment. Int J Radiat Oncol Biol Phys. 2005; 63: 1024–1028. 10.1016/j.ijrobp.2005.04.046 16005575 10.1016/j.ijrobp.2005.04.046

[15] Siker ML, Tome WA, Mehta MP. Tumor volume changes on serial imaging with megavoltage CT for non-small-cell lung cancer during intensity-modulated radiotherapy: How reliable, consistent, and meaningful is the effect? Int J Radiat Oncol Biol Phys. 2006; 66:135–141. 10.1016/j.ijrobp.2006.03.064 16839704 10.1016/j.ijrobp.2006.03.064

[16] Woodford C, Yartsev S, Dar R, Bauman G, Van Dyk J. Adaptive radiotherapy planning on decreasing gross tumor volume as seen on megavoltage computed tomography images. Int J Radiat Oncol Biol Phys. 2007; 69:1316–1322. 10.1016/j.ijrobp.2007.07.2369 17967322 10.1016/j.ijrobp.2007.07.2369

[17] Knap MM, Hoffmann L, Nordsmark M, Vestergaard A. Daily cone-beam computed tomography used to determine tumour shrinkage and localisation in lung cancer patients. Acta Oncol. 2010;49:1077–1084. 10.3109/0284186X.2010.498434 20831499 10.3109/0284186X.2010.498434

[18] Ramella S, Fiore M, Silipigni S, Zappa MC, Jaus M, Alberti AM, et al Local Control and Toxicity of Adaptive Radiotherapy Using Weekly CT Imaging: Results from the LARTIA Trial in Stage III NSCLC.J Thorac Oncol. 2017; 12(7):1122–1130. 10.1016/j.jtho.2017.03.025 28428149 10.1016/j.jtho.2017.03.025

[19] Duda RO, Hart PE, Stork DG. Pattern Classification (2nd Edition).Wiley-Interscience, 2000.

[20] Zhao G, Pietikainen M. Dynamic texture recognition using local binary patterns with an application to facial expressions. IEEE transactions on pattern analysis and machine intelligence 2007;29(6): 915–928. 10.1109/TPAMI.2007.1110 17431293 10.1109/TPAMI.2007.1110

[21] Saeys Y, Inza I, Larrañaga P. A review of feature selection techniques in bioinformatics. Bioinformatics 2007;23(19):2507–2517. 10.1093/bioinformatics/btm344 17720704 10.1093/bioinformatics/btm344

[22] Hua J., Xiong Z., Lowey J., Suh E. and Dougherty E. R., "Optimal number of features as a function of sample size for various classification rules," Bioinformatics, vol. 21, pp. 1509–1515, 2004. 15572470 10.1093/bioinformatics/bti171

[23] Kohavi Ron. "A study of cross-validation and bootstrap for accuracy estimation and model selection." Ijcai. Vol. 14 No. 2 1995.

[24] Agresti Alan. Categorical data analysis. Vol. 482 John Wiley & Sons, 2003.

[25] Mackinnon Andrew. "A spreadsheet for the calculation of comprehensive statistics for the assessment of diagnostic tests and inter-rater agreement." Computers in biology and medicine 30 3 (2000): 127–134. 10758228 10.1016/s0010-4825(00)00006-8

[26] Hanley James A., and Barbara J. McNeil. "The meaning and use of the area under a receiver operating characteristic (ROC) curve." Radiology 143 1 (1982): 29–36. 10.1148/radiology.143.1.7063747 7063747 10.1148/radiology.143.1.7063747

[27] Fleiss Joseph L., Levin Bruce, and Paik Myunghee Cho. Statistical methods for rates and proportions. John Wiley & Sons, 2013.

[28] Manrai Arjun K., et al "Medicine’s uncomfortable relationship with math: calculating positive predictive value." JAMA internal medicine 174 6 (2014): 991–993. 10.1001/jamainternmed.2014.1059 24756486 10.1001/jamainternmed.2014.1059 PMC4955674

[29] Coroller TP, Agrawal V, Narayan V, Hou Y, Grossmann P, Lee SW, et al Radiomic phenotype features predict pathological response in non-small cell lung cancer. Radiother Oncol. 2016;119(3):480–6. 10.1016/j.radonc.2016.04.004 27085484 10.1016/j.radonc.2016.04.004 PMC4930885

[30] van Timmeren JE, Leijenaar RTH, van Elmpt W, Reymen B, Oberije C, Monshouwer R, et al Survival prediction of non-small cell lung cancer patients using radiomics analyses of cone-beam CT images. Radiother Oncol. 2017;123(3):363–369. 10.1016/j.radonc.2017.04.016 28506693 10.1016/j.radonc.2017.04.016

[31] Antonia SJ, Villegas A, Daniel D, Vicente D, Murakami S, Hui R, et al Durvalumab after Chemoradiotherapy in Stage III Non-Small-Cell Lung Cancer. N Engl J Med. 2017;377(20):1919–1929. 10.1056/NEJMoa1709937 28885881 10.1056/NEJMoa1709937

